# Patterning, From Conifers to Consciousness: Turing’s Theory and Order From Fluctuations

**DOI:** 10.3389/fcell.2022.871950

**Published:** 2022-05-03

**Authors:** Thurston C. Lacalli

**Affiliations:** Biology Department, University of Victoria, Victoria, BC, Canada

**Keywords:** pattern formation, reaction-diffusion theory, irreversible processes, neurocircuit assembly, error suppression in evolution and development

## Abstract

This is a brief account of Turing’s ideas on biological pattern and the events that led to their wider acceptance by biologists as a valid way to investigate developmental pattern, and of the value of theory more generally in biology. Periodic patterns have played a key role in this process, especially 2D arrays of oriented stripes, which proved a disappointment in theoretical terms in the case of *Drosophila* segmentation, but a boost to theory as applied to skin patterns in fish and model chemical reactions. The concept of “order from fluctuations” is a key component of Turing’s theory, wherein pattern arises by selective amplification of spatial components concealed in the random disorder of molecular and/or cellular processes. For biological examples, a crucial point from an analytical standpoint is knowing the nature of the fluctuations, where the amplifier resides, and the timescale over which selective amplification occurs. The answer clarifies the difference between “inelegant” examples such as *Drosophila* segmentation, which is perhaps better understood as a programmatic assembly process, and “elegant” ones expressible in equations like Turing’s: that the fluctuations and selection process occur predominantly in evolutionary time for the former, but in real time for the latter, and likewise for error suppression, which for *Drosophila* is historical, in being lodged firmly in past evolutionary events. The prospects for a further extension of Turing’s ideas to the complexities of brain development and consciousness is discussed, where a case can be made that it could well be in neuroscience that his ideas find their most important application.

## Introduction

As graduate students in the early 1970s, we were aware of Turing’s reaction-diffusion theory of pattern formation, but it was at that time more a curiosity than a part of mainstream developmental thinking. Fifty years on, Turing’s ideas have been successfully applied to a number of developmental systems (Maini et al., 2006; 2012; Othmer et al., 2009; Kondo and Miura, 2010; Davidson and Baum, 2012; Chatterjee et al., 2020; Green, 2021), though the mechanistic details often differ from his original proposal, with chemical autocatalysis being replaced by other self-enhancing molecular or cellular processes, and distance effects by other means of material transport, or by mechanochemical effects (for the latter, see Murray and Oster, 1984; Howard et al., 2011; Brinkmann et al., 2018; Veerman et al., 2021). For theorists, there have been disappointments along the way, in that patterns that appeared to match theoretical prediction were shown to arise by other mechanisms. But despite this, the theoretical enterprise has now reached a healthy middle age, with expectations of a vigorous and productive future. This review is designed as a broad survey with a focus less on mechanistic details than on what I consider the main turning points in the acceptance of Turing’s insight regarding the kinetic basis of pattern selection, whether specifically by reaction and diffusion, or via other means of self-enhancement and action over distance. While my account of the subject is retrospective, the intent is not, as my interest is in part to consider how Turing’s ideas might be extended in future, notably to neuroscience, as a way of accounting for the precise construction of the neurocircuitry required to support consciousness in the brain (Lacalli, 2020; Lacalli, 2021). Among scientific problems in search of a solution, this must surely be among the most daunting, and it remains a distinct possibility that the acknowledged importance of Turing to computer science (De Mol, 2021) will be equaled or surpassed in biology should his ideas on patterning prove applicable to the problem of biological consciousness.

## Early Days

**FIGURE 1:**
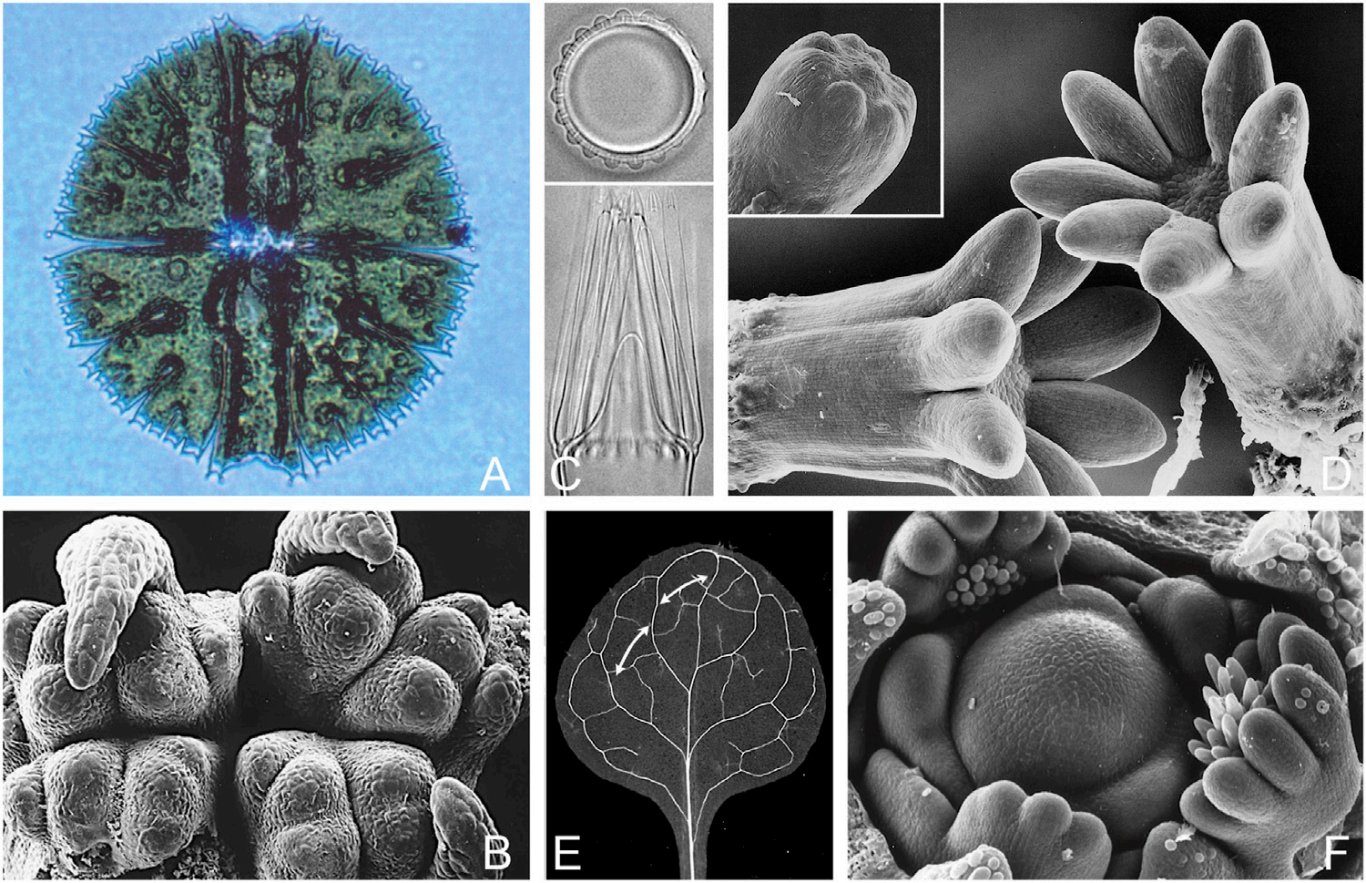
Turing-type patterns in plants: branching and whorl formation in uni- and multicellular examples, and leaf venation. **(A)**. Dichotomous branching in one dimension: the freshwater desmid *Micrasterias rotata*, (cell diameter 230 μm) where form is generated following cell division by branching tip growth along the edge of the expanding semicell. The effective pattern scale (wavelength) declines during this process from ca. 30 μm at the beginning to 5 μm for the distal branches; see Lacalli & Harrison (1987) for quantitative details. **(B)**. Alternating dichotomous branching in two dimensions: the shoot apex of *Psilotum nudum*, a basal fern whose simple aerial shoots originate through repeated dichotomous branching and only elongate, together, secondarily. Distance between adjacent primordia at this stage is in the 150–200 μm range (Takiguchi et al., 1997), but the pattern wavelength has not been measured through the branching sequence, and could well vary; specimen supplied by T. A. Steeves. **(C)**. Whorl formation in a single cell: the pattern of hair initials (top) and their outgrowth (bottom) in the dasyclad alga *Acetabularia*. The distance between initials, typically 20 μm in culture, can range between 16 and 28 μm in a predictable way depending on temperature and calcium concentration, and from this one can make useful inferences about the mechanism; see Harrison & Hillier (1985), Dumais & Harrison (2000) for details. **(D)**. Whorl formation in conifers: the cotyledons (primary needles) of cultured white spruce embryos; stem diameter is ca. 750 μm compared with 400 μm when the initials are first evident (inset), with a spacing of ca. 95 μm (Fowke et al., 1994). The most detailed statistical information available on cotyledon spacing is for larch, where the pattern wavelength has been measured precisely, at 98 ± 4 μm (Harrison & von Aderkas, 2004; Holloway et al., 2018). **(E)**. Leaf venation in a young *Arabidopsis* leaf, where distance between secondary veins (arrows) in part reflects a spacing mechanism that acts along the leaf margin as the primordium develops. The leaf blade is ca. 2 mm long at the stage shown, but the first secondary veins appear when it is 20-fold smaller (100–120 μm long) with an effective wavelength between secondaries as they develop in the 20 μm range, down to a few cell diameters (10–15 μm) in some instances (Scarpella et al., 2006, Wenzel et al., 2007, Verna et al., 2019; see Holloway & Wenzel, 2021 for relevant modeling). The mechanistic basis of the discrepancy between vascular patterning at this scale and that of primordia across the apical meristem is as yet unresolved. **(F)**. The shoot apical meristem of lupin (*Lupinus polyphyllus*), one of the largest among the angiosperms, with a central dome ca. 250 μm across at its base. The overall pattern of primordia, typical of angiosperms (with some exceptions, e.g., of decussate pattern), is one of spiral phyllotaxy, but the leaves are palmate, developing as partial whorls as can be seen here in three examples, where spacing would appear to be on a scale somewhere below 30 μm; see Runions et al. (2017) for a further discussion of leaf shape in relation to spacing mechanisms acting along the leaf margin. Photo credits: (A, B) T. C. Lacalli, (C) Jacques Dumais, (D) L. C. Fowke, (E) Enrico Scarpella, (F) V. K. Sawhney.

The publication of Turing’s paper on morphogenesis (Turing, 1952) resulted in a brief period of interest among biologists, due in part to the efforts of C. W. Wardlaw, then Professor of Cryptogamic Botany at Manchester University (Wardlaw, 1953). Wardlaw would have been familiar with diverse examples of whorl formation and dichotomous branching in algae, ferns and the like, and that comparable patterns occur at both the unicellular and multicellular level (Figure 1). This latter feature probably accounts, at least in part, for the greater willingness of developmental botanists, in contrast to their zoological counterparts, to consider pattern as an entity in its own right irrespective of mechanistic details. So, for example, one can study whorl formation in a single cell, like the dasyclad alga *Acetabularia*, where distance effects are likely due to diffusion (Dumais et al., 2000), or in the apical meristem of conifers (Harrison and von Aderkas, 2004), where distance effects arise through polar transport of auxin between cells (Reinhardt et al., 2003; Shi and Vernoux, 2019). Auxin transport can also produce patterns suitable for explaining both phyllotaxis (Jönsson et al., 2006) and leaf veins (Mitchison, 1981; Scarpella et al., 2006; Biedroń and Banasiak, 2018) where, for the latter, regularity of spacing along the leaf margin appears to play an important role (Holloway and Wenzel, 2021; Lavania et al., 2021). Yet for all these examples, the mathematical and computational problems of dealing with material flow, growth and mechanistic redundancy will be much the same.

**FIGURE 2:**
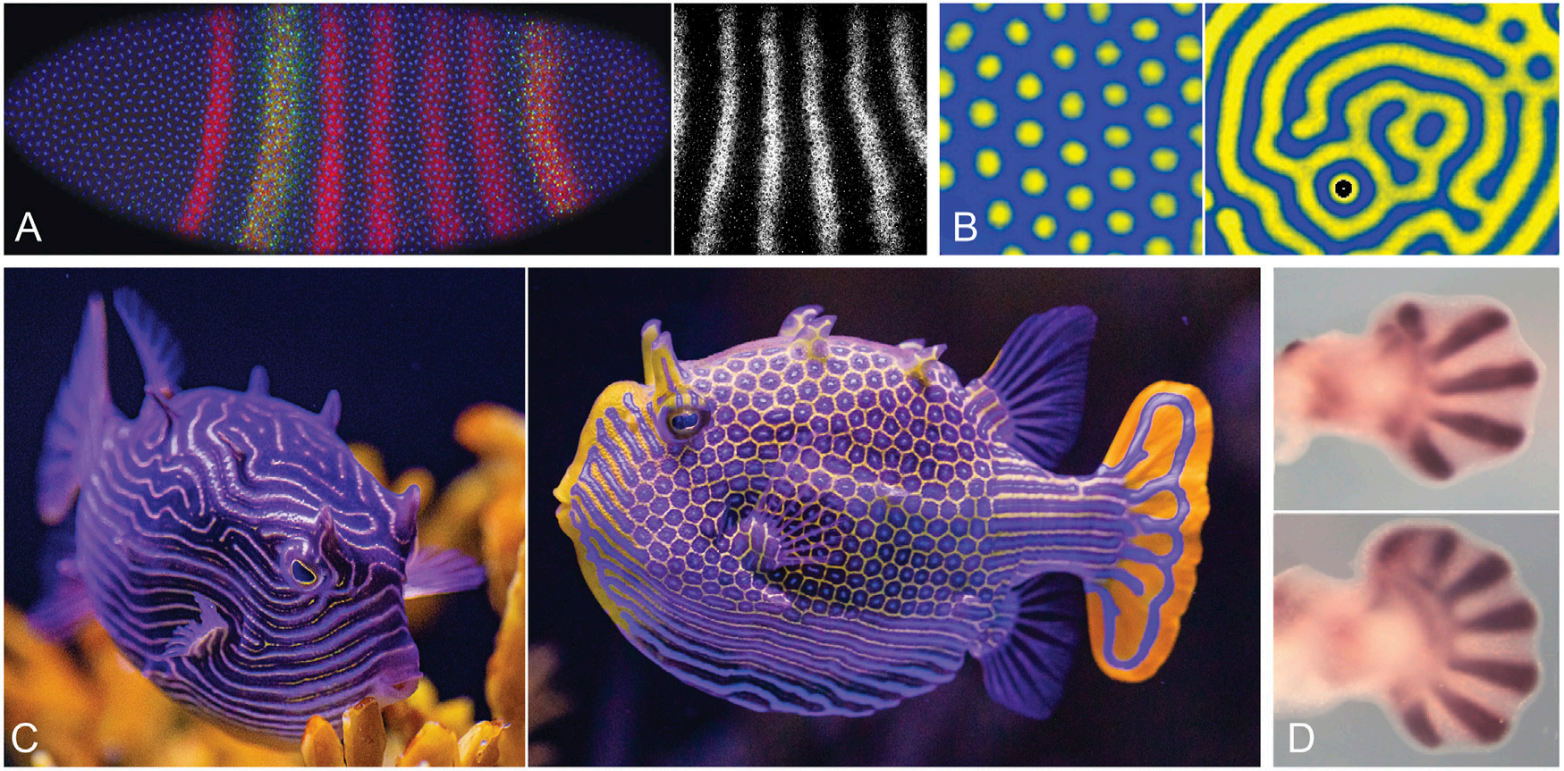
Selected animal and chemical patterns: stripes, spots, and digits. **(A)**. The *Drosophila* pair-rule pattern. Left: an embryo at stage 5 (length 505 μm, anterior to the left), nearing the completion of cellularization; nuclei in blue, even-skipped (*eve*) protein in red, with an enhancer tag (green) showing specificity for some stripes rather than others, a clear demonstration of stripe-specific control over *eve* expression. Right: detail of the *ev*e transcript pattern; stripe spacing (centre-to-centre distance) is ca. 40 μm. **(B)**. Chemical patterns, showing arrays of spots (left) and labyrinthine stripes (right) produced by the TuIS (thiourea-iodate-sulfite) reaction in a gel medium, a variant of the better known CIMA reaction. Spacing between pattern elements is ca. 2 mm; see Horvath et al. (2009) for details. **(C)**. The ornate boxfish, *Aracana ornata*, native to waters off South Australia; female (left) and male (right) showing mixed stripe and spotted patterns characteristic of boxfishes, which often vary between the sexes despite, presumably, a common underlying mechanism. **(D)**. Digit development in mouse embryos, showing patterns of the marker *Sox9* in wild type limb (top) and the expanded fan of digits produced by the homozygous *Gli3* null mutant (bottom). The pattern here is realized as a series of cartilaginous elements, but is a result of a one-dimensional periodicity along the limb margin that lays down a two-dimensional pattern as the limb grows (Hiscock et al., 2017), a 1D to 2D transition comparable to that seen in *Micrasterias*. The number of digits increases further in *Hox11/13* mutants, but the underlying pattern results from Turing-type interactions between the protein products of *Bmp*, *Sox9* and *Wnt* genes; see Raspopovic et al. (2014) for details; Onimaru et al. (2016), Stewart et al. (2017), Newman et al. (2018) for evolutionary perspectives. Photo credits: (A, left) Thomas Gregor, (A, right) Erik Clark, (B) Istvan Szalai, (C) the Birch Aquarium at Scripps, (D) Rushikesh Sheth and Marian Ros.

Developmental zoologists, faced with a more diverse range of patterning situations, have tended to focus more on identifying the proximate causal agents in each case than on the general features of broadly based theories like Turing’s. And in any case, the conventional wisdom in the early days, expressed by C. H. Waddington (see Waddington, 1956; page 423), was that a reaction-diffusion mechanism, being “inherently chancy” could at most account for the dapplings and mottlings filling otherwise unimportant spaces. It did not help that the specific equations Turing devised did not always produce regular patterns (Bard and Lauder, 1974), or that the one 2D pattern Turing included in his 1952 paper (his Figure 2) was itself rather irregular. But that example was computed for what Turing himself considered the least interesting case [his case *(a)*, stationary waves of moderate wavelength being case *(d)*], and his preliminary attempts to document the formation of regular 2D patterns were unpublished at his death (Dawes, 2016). Hence, by default, it was left largely to physical chemists to explore Turing’s ideas more fully, and the energy-dissipative, far-from-equilibrium thermodynamics they embody. This was carried forward initially by Illya Prigogine and his Brussels research group (Prigogine and Lefever, 1968; Nicolis and Prigogine, 1977), using a hypothetical reaction system, the Brusselator, that was subsequently widely used and adapted by others (Tyson and Light, 1973; Harrison, 1987; Subramanian and Murray, 2021). On the experimental side, there was increasing interest in the Belousov-Zhabotinsky reaction, renowned for the production of oscillations and moving waves (Field and Burger, 1985; Zhabotinsky, 2007), a phenomenon so striking at the time as to be met frequently by disbelief among chemists when first encountered. This led, on the theoretical side, to an interest in model reaction systems that produced periodic oscillations that could be used to model biological processes, notably circadian rhythms (Winfree, 1980), segmentation (Cooke and Zeeman, 1976; Newman, 1993; Pourquié, 2003), and the mitotic oscillator, where Tyson has continued to make important contributions (e.g., see Tyson and Novák, 2015).

Interest in Turing’s ideas was reignited among developmental zoologists by two developments, first the work of Gierer and Meinhardt (1972) modeling pattern formation and regeneration in hydra, and second, by Murray’s comparatively well-received (for theory) account of animal coat patterns (Murray, 1981; Murray, 1988). Neither of these efforts, however, led immediately to a reassessment of the relevance of Turing’s theory to a wider range of developmental examples. This was perhaps in part because a role in specifying coat patterns reinforced the existing notion that the theory was applicable only to irregular surface patterns, but also because the Gierer-Meinhardt model, designed to amplify an existing prepattern rather than generating well-controlled patterns *ab initio*, was not at first widely recognized for being a Turing model in disguise. There was also, for a time, a degree of suspicion verging on hostility towards Turing’s theory on the part of some proponents of other mechanisms, where it should have been obvious from the start that Turing-type mechanisms could well be acting in concert with, for example, position-specific signaling, but at a different stage in the patterning process, as has proved to be the case (Miura, 2013; Green and Sharpe, 2015; Newman et al., 2018).

Even so, the main impediment to wider acceptance of Turing’s ideas among biologists has always been, and remains, a matter of expectations: that theory was to be judged in strictly reductionist terms, as to whether it either does, or does not provide a route towards identifying the proximate entity responsible for the pattern in question, be this a gene, a diffusible morphogen, or something else. This is different from the biomathematical focus, towards anything in biology that yields interesting mathematics, and from preconception of the physical chemistry community, that progress is first and foremost a matter of understanding principles and process, a point of view well represented in Harrison’s account of the subject (Harrison, 1987; Harrison, 1993). Here the details matter less than identifying the range of possible classes of explanation and establishing ground rules for distinguishing between them. Harrison identifies three such classes, of kinetics, self-assembly, and equilibrium, where Turing’s model belongs to the first. But among the broad class of kinetic processes, the subset of importance to patterning are those able to act as selective amplifiers, extracting a signal from the statistical noise of real-time molecular behavior. This is in fact the essence of Turing’s conception, explicit in the form of the solutions, and it precisely on this point, the form of the solutions, that he begins his mathematical account (Turing, 1952, pg. 39). The issue of the sensitivity (i.e., instability) of the un-patterned, homogeneous situation to fluctuations is then raised at various points in the text (e.g., pp. 56–57), using oscillatory electrical circuits as a point of reference. It is this feature I want specifically to highlight as distinguishing Turing’s theory (here Turing-type models or, in Harrison’s usage, kinetic theory and the kinetic preconception more broadly, or the “Turing problem” referred to by Kang et al., 2012), from other ways of accounting for biological pattern. And, for macro-scale biological pattern, kinetic mechanisms with the properties described by Turing would seem to have a distinct edge: “but what else could do it?” Harrison quotes a colleague as saying. The question here is rhetorical, and I return to it below (see section *Inelegance and Ratchets, Error Suppression and Time*) because, when it is indeed something else that “does it”, that something else needs to be characterized and understood. Applying the order-from-fluctuations principle more generally, there are three things to consider when distinguishing classes of patterning models in terms of what they do and how they do it: the nature of the fluctuations, the identity of the amplifier, and the time scale on which these both operate, where more than one notion of what we mean by “time” may be required.

## Flexibility in Pattern Selection: Spots, Stripes and In-Between

Of various inaccurate notions about Turing’s theory, the first that needs addressing is Waddington’s objection, that it accounts only for irregular dapplings and mottlings. To do more than this, what is needed is a mechanism that is sufficiently flexible in the patterns it produces that it can be adapted by evolution as required. So, for example, on a two-dimensional surface the Gierer-Meinhardt model produces an irregular array of peaks frozen in place, which is not particularly useful for producing regular patterns of spots or stripes, let alone anything more elaborate. But the Gierer-Meinhardt model is rather idiosyncratic in this respect, because other models, including the Brusselator, generate regular hexagonal arrays of spots with ease. Turing’s own notes show preliminary calculations approaching this result, where there were parallels with contemporary observations in fluid dynamics (Dawes, 2016), but the first fully developed computational examples using reaction-diffusion equations, so far as I am aware, came from my own work on pattern in unicellular algae (Lacalli, 1981). The ability to produce a modulated, well-controlled pattern in two and three dimensions means also the ability to respond to changing influences throughout the non-linear phase of pattern development, including boundary conditions, imposed gradients and the presence of neighboring pattern elements (Hiscock and Megason, 2015), as well as the ability to subdivide cell and tissue domains in an orderly way (Lacalli and Harrison, 1978; Hunding, 1984). In stark contrast to Waddington’s view, and depending on the mechanistic details, boundary conditions and the like, a Turing model can in principle produce almost any pattern one cares to choose, and will do so in a reliable and reproducible fashion: “bespoke” patterning to borrow a phrase from Woolley et al. (2021), with evolution as the customer.

The ability to generate orderly patterns of stripes, in particular, quickly became a focus of attention with the discovery of the pair-rule pattern that precedes the formation of morphologically distinct segments in *Drosophila* embryos (Hafen et al., 1984 for the *fushi-tarazu* gene; see also Pick, 2016). The precision of this pattern at the cellular level (Figure 2A), with multiple stripes appearing essentially simultaneously, was astonishing at the time, and was interpreted by some, including myself, as strong circumstantial evidence for the involvement of a kinetic mechanism. The link between Turing and *Drosophila* stripes proved to be a bridge too far, as position-specific molecular events involving complex assemblages of transcriptional modulators responsive to graded signals along the length of the embryo were soon thereafter shown to be the means by which pattern was specified (Štanojević et al., 1989; Struhl et al., 1989, Struhl et al., 1992; Rivera-Pomar and Jäckle, 1996). This has been characterized as an inelegant solution to the patterning problem (Akam, 1989), in contrast to simplicity of global control over pattern, with pattern landmarks being preset by the action of maternal and gap genes (Diaz-Cuadros et al., 2021). A greater degree of hierarchical control is thereby imposed over what was previously, in more basal arthropods, a self-organizing process of sequential segmentation relying, as in vertebrates, on molecular clocks and moving wavefronts (Salazar-Ciudad et al., 2001; Pourquié, 2003; Hunding and Baumgartner, 2017). A sequential mode of segmentation is characteristic of basal arthropods and short germ band insects like the beetle *Tribolium*, and there are plausible scenarios for linking this to the *Drosophila* condition through a complicated series of transitional steps (Clark et al., 2019; Clark, 2021). If there is elegance here, it is well hidden.

Even though the *Drosophila* pair-rule pattern proved not to depend on a Turing-type mechanism, the striking regularity of the pattern was a significant spur to theorists to understand the conditions under which model systems would generate stripes as opposed to spots or other patterns, in other words, to define the rules for pattern selection. This was first addressed in two nearly simultaneous publications, by Lyons and Harrison (1991) and Ermentrout (1991), making it immediately clear why symmetry features of the non-linear phase of pattern development are important, in that matched positive and negative departures from the steady state favored stripes (Lyons and Harrison, 1992). Further fueled by interest among chemists in the CIMA reaction (Lengyel and Epstein, 1991; Abdelmalek and Bendoukhu, 2020), which forms regular arrays of spots, stripes, and intermediate reticulate or labyrinthine patterns (Figure 2B), a burgeoning experimental literature appeared on pattern in chemical reaction systems (e.g. Ouyang and Swinney, 1991; Boissonade et al., 1995; Konow et al., 2021), with parallel advances in the theory (e.g. Dufiet and Boissonade, 1992; De Wit, 1999; Cross and Greenside, 2009). On the biological side, striking observations on fish pattern by Kondo and Asai (1995) made the likely involvement of a Turing-type mechanism of some kind increasingly hard to deny. And, while fish patterns arise through dynamics operating at the cellular level rather than diffusing reactants (Kondo et al., 2021), this does not matter when the point of the exercise is to validate the theory for kinetic processes as a class. Zebrafish have proven a useful model system here as well (Singh and Nüsslein-Volhard, 2015; Kondo et al., 2021), and even more dramatic patterns, combining arrays of spots, stripes and reticulate intermediates, are seen in coral reef fishes, amongst which boxfishes are noteworthy examples (Figure 2C; see Pearson, 1993, and Othmer et al., 2009 to compare with a range of computed examples). Combining these observations with more recent work on digit patterns (Figure 2D; see Newman and Frisch, 1979; Sheth et al., 2012; Raspopovic et al., 2014; Newman et al., 2018; and Chatterjee et al., 2020 for the basic theoretical case), it appears that two of the main objections to Turing’s ideas as applied to animal systems have been answered, at least for vertebrates, that 1) kinetic theory is perfectly capable of explaining a range of surface patterns that are regular, highly controlled and flexible in their adaptive capabilities, and 2) not only surface pattern, but skeletal patterns lodged within the body depend at least in part on Turing-type mechanisms (see Painter et al., 2021 for other examples of internal patterning).

A final, perennial objection to Turing’s reaction-diffusion mechanism is a supposed lack of robustness, that pattern formation depends on the parameters being adjusted within a narrow range. While this is true to a degree of 2-component models, more recent work has shown that having more components, especially if some are non-diffusing (Marcon et al., 2016; Diego et al., 2018; Landge et al., 2020; Krause et al., 2021), and discrete rather than continuous systems (Leyshon et al., 2021), yields models far more robust than previously supposed possible, and there is now a better understanding of how pattern stability is maintained in the non-linear regime (Subramanian and Murray, 2021). The burden of past misconceptions concerning kinetic theories has thus now, in large part, been removed.

## Inelegance and Ratchets, Error Suppression and Time

The idea of the inelegance of the mechanisms underlying developmental pattern captures both a superficial truth and a deeper one. On the one hand, inelegance in this context refers to the complexity of developmental phenomena at the molecular level, which verges on the illogical (Lewin, 1984). Elegance equates to simplicity, in that patterning by a Turing-type mechanism can be encapsulated in a few lines of mathematical symbols, whereas accounting for the pair-rule pattern requires a detailed inventory of molecular components and their myriad functional interactions. But while the *Drosophila* stripe issue was resolved largely in favor of inelegance, the failure of theory, as often in science, proved a more interesting and informative result than success. In this instance, it led to a new appreciation of the problem of achieving a reliable developmental result in the face of the random noise that characterizes molecular events in the real world (Rao et al., 2002; Balázsi et al., 2011). The question was first posed in theoretical terms (Holloway and Harrison, 1999; Kang et al., 2012), and then addressed experimentally in considerable detail using *Drosophlia*, initially in work carried out by Eric Wieschaus and collaborators (e.g., Houchmandzadeh et al., 2002). This was part an emerging trend that has since made biomolecular science more quantitative (Maddox, 1992; Davidson and Baum, 2012; Gregor et al., 2014), and there is now both a much increased appreciation of the importance of error suppression in developmental systems at the molecular level, with the production of the *Drosophila* pair-rule pattern as a key model (Petkova et al., 2019; Bauer et al., 2021), and a far better understanding of how this is achieved.

Conceptually, the questions that need addressing, of precision, reliability and robustness, are more general than any one example, or any one pattern. And, if a Turing-type mechanism is not involved, we return to the chemist’s question, above, but now applied to error suppression: “but what else could do it?” The answer from *Drosophila* is that we have left the realm of microscopically reversible kinetic processes, where Turing models reside, but neither is this structural self-assembly of a jigsaw-puzzle type, e.g., of a virus particle. Instead, the *Drosophila* pair-rule pattern depends on macromolecular complexes that decode and implement a set of genetic instructions, and are assembled in a series of steps that are, in effect, thermodynamically irreversible (Carey, 1998; Poss et al., 2013). At the level of transcriptional control, this involves multiple enhancer elements that act at the level of the gene in a combinatorial way to optimize the response to graded inputs that convey information on cell position (Chen et al., 2018; Furlong and Levine, 2018; Petkova et al., 2019; Bauer et al., 2021), but there are strategies at all levels of the process, from the shape of the gradients (Song and Hyeon, 2021) to mechanisms for sharpening the stripes (Munteanu et al., 2014), that have been likewise optimized by evolution to ensure that patterning proceeds in a way that minimizes errors. To emphasize the programmatic aspect of the molecular assembly part of the process, I suggest the term “programmatic assembly”, which is also ratchet-like, to use a mechanical analogy (Oster, 2002), while being both combinatorial and synergistic, and there is a graph-theoretical formulation, of micro-states linked by unidirectional edges representing the irreversible assembly steps (Ahsendorf et al., 2014; Martinez-Corral et al., 2021) that is especially promising as an analytical methodology going forward. Implicitly all such approaches face the same problem, that, to quote from Ahsendorf et al., “history cannot be ignored away from thermodynamic equilibrium”, where by history, we mean the sequence of steps by which the machinery in question is assembled and operates. But there is a second history, and a second time scale, of the evolutionary sequence by which the machinery itself was refined and perfected over many generations, with all the contingency that implies. Taking the molecular level equivalents of coding and decoding as an example (e.g., Jarzynski, 2008; 2011), fully accounting for the thermodynamic driving forces behind each step in such cases is a complex and sometimes counterintuitive exercise. The same is true at a more abstract level, for a concept like positional information, since a device able to read and interpret such information will necessarily, like a human reader, be an energy dissipative product of evolution operating irreversibly far from equilibrium.

To go yet further, to the level of physics, the issue becomes one of time, of whether, in the terminology of Cortês and Smolin (2014), one is dealing with passive time or generative time. Passive time in this context is the “t” that appears in a typical set of equations, whether for Turing’s mechanism or for calculating a ballistic trajectory, and solving such equations yields the same answer each time. *Drosophila* segments also form the same way each time, but there is a difference. To see this, consider error suppression yet again, and how a developmental outcome can be produced as precisely realized as a pair-rule stripe. For a Turing mechanism, error suppression depends on feedback steps in the mechanism that amplify fluctuations and, together with diffusion, select one pattern over all others, including over background noise, doing so in real time as the pattern develops. For the transcriptional machinery employed in *Drosophila* segmentation, in contrast, the feedback step is evolution itself, in its role as a generator of gene sequences for the enhancers and transcriptional regulators required to produce the pattern in question, and to suitably refine their interactions. So in this case error suppression is in large part historical, that is, *it has already occurred*. And, because it is then embedded in the codes and structures that implement the genetic program, it does not appear explicitly in equations that model change in real time. Similarly, if we think about the fluctuations on which the amplifier acts, for a programmatic assembly process these are not spatial in character, but arise from genetic variation at the population level, because different individuals will vary as to the precision with which they replicate pattern, and it is by eliminating the more error-prone individuals, generation by generation, that the genome evolves in ways that reduce developmental errors for the population as a whole. It is then this mix of time scales and of history-dependent and history-independent features, which in analytical terms must be dealt with separately, rather than complexity *per se*, that precludes an elegant solution. From an error-suppression standpoint, this means that the problem of statistical noise at the level of positional cues can be dealt with analytically in a straightforward way (as by Tkačik and Gregor, 2021), but reliability and accuracy at the level of the interpreter cannot, as the evolutionary steps by which that interpreter was conjured into existence are inescapably part of the story. This also means, for the experimentalist, that quantitative tests of reliability for examples of programmatic assembly are less a measure of the physical limits of a given class of mechanisms, than they are of the effectiveness of evolution in its choice of an error-suppression strategy for each step in the assembly process.

A further lesson from *Drosophila* is, or would seem to be, that where evolution has replaced one mechanism by another, the transition is more likely than not to be in the direction of increased reliance on programmatic control, so that development becomes more complex, and hence inelegant, over time. For *Drosophila* in particular, the proximate advantage of making this change can be measured in the developmental time saved, as segment specification is significantly faster in *Drosophila* than in basal arthropods and short germ-band insects. This is a distinct advantage for insects like fruit flies, whose larvae compete with fungi and nematodes for a rapidly depleting food resource. But there is a potential cost in the loss of one key feature of oscillatory, clock-based segmentation mechanisms, in that errors accumulated from past steps in the developmental program are no longer overwritten by the new pattern and reset to zero. That this cost is not paid in reduced developmental reliability in *Drosophila* shows that programmatic assembly solves the problem of error suppression by other means, namely through structural innovations and enhanced specificity in the molecular machinery that implements the developmental program. This then begs the question of whether this same solution has been employed in the past, perhaps repeatedly, in multiple development pathways as a means of speeding the overall process of embryogenesis. Germ layer specification, for example, depends on highly complex gene regulatory networks (Loose and Patient, 2004; Kiecker et al., 2016), and is hence a good candidate for having imposed a programatic overlay on simpler, more purely kinetic ancestral mechanisms in order to achieve the same result more rapidly. There are implications here also on the botanical side, in providing a rationale for why mechanisms for plant patterning are generally more conserved across taxa than is typical of animals: that growth and patterning are tightly integrated in plants (Dumais and Kwiatkowska, 2002; Harashima and Schnittge, 2010; Rebocho et al., 2017), and so long as it is growth rather than patterning that is rate-limiting, there is little to be gained by reducing the time required to specify pattern. Ancestral mechanisms are then more likely to be retained rather than being replaced.

## Conclusions, and Some Final Thoughts, on Thought

It has been gratifying, over the last 2 decades, to see Turing’s ideas gaining acceptance and proving their worth in specific biological situations. But this is only part of a larger enterprise, in the past a concern mainly of the more physico-chemically minded, but now more widely recognized, which is to better understand the essential underlying features of kinetic mechanisms as a class. A key concept here, featured in Turing’s own account, is the idea of generating order from fluctuations, that is, of extracting a meaningful signal from the underlying noise of the system, which can be at a molecular, subcellular or cellular level. The issue has relevance across a range of examples: from purely chemical systems, such as the CIMA reaction, to hybrid ones, like *Drosophila*, relying more on programmatic assembly than simple kinetics. Examples of programmatic assembly are then inherently less elegant than purely kinetic mechanisms because real time events play a lesser role than evolutionary ones. So, for example, achieving a precise outcome reliably depends on processes unfolding largely in real time for a kinetic mechanism like Turing’s, but for programmatic assembly these are embedded in the past, in the evolutionary sequence that produced the machinery that executes the program. Programmatic assembly cannot therefore be fully understood except in the context of an extended sequence of evolutionary events, which begs an analytical question, of how to deal in practice with events unfolding in two mutually exclusive time scales.

A final point I want to address is whether we have been missing what is potentially the most important application of Turing’s ideas, to controlling the assembly of neural circuits in the developing brain. If we consider the various cellular level activities needed to correctly configure the neural circuitry underpinning complex brain functions, there are many opportunities for competitive dynamics of the kind envisioned by Turing, but played out at a structural level, of cells, synapses and dendrites, rather than diffusing molecules (Lacalli, 2020). Turing himself had considered this issue, as is evident from a letter to J. Z. Young in February of 1951 (see Hodges, 1983, pg. 436), and his ideas have potential application to the period of synaptic remodeling that occurs in the neonatal nervous system, including in the cortex, whereby excess neurons and synaptic connections are removed in an activity-dependent way in response to sensory feedback (Le Bé and Markram, 2006; Low and Cheng, 2006; Kano and Hashimoto, 2009). But this is also the period when the newborn begins to develop a conscious awareness of its surroundings (Lagercrantz and Changeux, 2010), and for the circuits responsible for the basic sensations of phenomenal consciousness, i.e. qualia in most formulations, there is a problem. To illustrate this, consider a newborn hearing a sound, or experiencing pain, for the first time. The problem here is the absence of feedback mechanisms to correct any errors that may occur in the quality and character of the sensation evoked by the neural circuitry to which this task has been assigned. In other words, if the circuits evoking a particular sensation, of pain for example, or sound or light, have been incorrectly assembled in the embryonic period, the resulting sensations, whether they are the correct sensations or not, simply become the nature of experience for that individual. The brain thus faces the same problem that an insect does in correctly forming its segments, that it has one chance to get it right. The developmental options for doing so should then also be the same: to develop in a programmatic way to yield what is essentially a deterministic result, as in *Drosophila*, or to instead employ a Turing-type process of dynamic competition, either during the initial phase of circuit development or later remodeling, to amplify some circuitry variants at the expense of others. There may in fact be no single answer, as mechanisms by which brain circuitry is assembled will undoubtedly vary across taxa, from being more programmatic in the brains of small rapidly-developing invertebrates, to less programmatic in the brains of larger animals showing more flexible modes of learning and behavior, most notably cephalopods and vertebrates. For the circuits responsible for consciousness more specifically, there could in fact be a sequence, similar to that in insect segmentation, with global kinetic mechanisms being the ancestral way of generating the circuits responsible for phenomenal sensations as these first emerged in evolution, with more streamlined, programmatic ways of achieving the same result evolving secondarily.

We have, in sum, three options as to how the neural circuitry responsible for conscious sensation is assembled: that 1) it originated and remains a product of a global Turing-type patterning system operating at a structural, neurocircuitry level, or 2) like insect segmentation, it began that way but has since been converted, as in *Drosophila*, to some form of programmatic assembly, or 3) that the efficiencies inherent in programmatic assembly were themselves an essential part of the ability to evolve consciousness in the first place. It may be a mammalian bias to suppose that flexibility in behavior depends on more flexible, non-programmatic modes of development than is typically encountered in small invertebrates like *Drosophila*, but the general point remains valid in any case: that there are multiple scenarios under which mechanisms like those devised by Turing would lie at the very root of consciousness, and hence of the abilities of members of our species to engage in such activities as meaningful speech, logical thought and, not least, formulating and solving equations like Turing’s.
